# Current Status of Photodynamic Therapy in Digestive Tract Carcinoma in Japan

**DOI:** 10.3390/ijms16023434

**Published:** 2015-02-04

**Authors:** Atsushi Nanashima, Takeshi Nagayasu

**Affiliations:** Department of Surgical Oncology, Nagasaki University Graduate School of Biomedical Sciences, 1-7-1, Sakamoto, Nagasaki 852-8501, Japan; E-Mail: nagayasu@nagasaki-u.ac.jp

**Keywords:** photodynamic therapy, digestive tract cancer, bile duct carcinoma, porfimer sodium, talaporfin sodium, Japan

## Abstract

Photodynamic therapy (PDT) is an effective local treatment modality as a cancer-specific laser ablation in malignancy of some organs including digestive tracts or bile duct. In Japan, PDT has been applied at the early period after the first clinical induction in 1980’s. Although the useful efficacy was clarified, PDT has not been fully applied because of the phototoxicity of the porfimer sodium. The next generated talaporfin-sodium was used for PDT, in which phototoxicity was reduced and, however, the clinical efficacy for digestive tract malignancy has not yet been clarified. By proceeding the experimental and clinical trials, it is necessary to clarify the evidence of efficacy as a local powerful treatment with the conventional surgery, brachiotherapy and chemotherapy in the future step.

## 1. Introduction

### Induction of Photodynamic Therapy for Digestive Tract Carcinoma in Japan

Photodynamic therapy (PDT) has been clinically applied for human carcinomas by Dougherty* et al.* [1] since 1978. Since the 1990s, there have been many advances of PDT in the treatment of digestive tract carcinomas, such as in the esophagus, stomach, and bile duct, in Japanese series [2,3,4,5,6,7,8]. In these series, PDT using 2 mg per kg body weight of porfimer sodium (Photofrin^®^; Wyeth-Takeda, Tokyo, Japan) was used as a photosensitizer. After administration, patients avoided direct sunlight for 4–6 weeks. The excitation light source used was an Excimer dye laser (PDT-EDL1: Hamamatsu Photonics K.K., Hamamatsu, Japan). The wavelength was 630 nm, irradiation output was 4 mJ/pulse/cm^2^, and the frequency was 40 Hz. Irradiation was performed with the free cut or cylindrical type of laser fiber placed approximately 1 cm from the lesion to make an irradiation spot of 1 cm^2^ at 48–72 h after photosensitizer administration. Light doses consisted of 100 J/cm^2^. By the above investigators’ efforts, PDT has been approved as a radical treatment in the digestive tract for superficial carcinoma of the esophagus and stomach under the national health insurance system by the Ministry of Health and Welfare, Japan, since 1994.

## 2. Results and Discussion

### 2.1. Photodynamic Therapy (PDT) for Esophageal and Gastric Carcinoma

PDT using porfimer sodium (P-PDT) was shown to be an effective and radical treatment option for superficial esophageal and gastric adenocarcinoma; however, the use of this modality has not spread clinically because of the need for long-term shielding from light and an expensive laser apparatus. Endoscopic mucosal resection (EMR) and endoscopic submucosal dissection (ESD) were developed for minimally invasive, organ-sparing endoscopic removal of early malignant lesions in the gastrointestinal tract worldwide, including in Japan, during the same period [9,10,11]. In the case of a contra-indication for EMR or ESD, argon plasma coagulation could also be applied at a lower cost than P-PDT [12]. Therefore, the P-PDT option is currently excluded from the Japanese guidelines for the treatment of gastric cancer and P-PDT has only been performed at limited Japanese institutions.

### 2.2. PDT for Bile Duct Carcinoma

With respect to other digestive tract carcinomas, P-PDT was clinically applied for unresectable bile duct carcinomas worldwide, including in Japan. Between 1998 and 2006, European cancer institutes showed the improved patient status and survival benefits of P-PDT for unresectable bile duct carcinomas by randomized or phase II prospective trials [13,14,15,16,17,18]. The most recent articles on PDT for unresectable BDC indicated improvement in liver function, patient quality of life, and prolongation of the survival period, with few complications [19]. Thus, PDT also appears to be a promising modality for local treatment in BDC. A few Japanese institutes have shown the effectiveness of P-PDT via case reports and a retrospective study reported in English [5]. P-PDT was also described as being effective for prolonging the survival period in resected bile duct carcinoma in German reports [20,21]. Our report also showed the effect of P-PDT for the local control of bile duct carcinoma in unresectable cases and resected cases with remnant microscopic cancer cells at the bile duct stump [22]. At our institute, departments of digestive medicine, digestive and thoracic surgery, oral surgery, gynecology, dermatology, and brain surgery have attempted PDT in each field of cancer treatments; thus, Nagasaki conferences specializing in PDT have been held since 2004. Unfortunately, the maintenance service of PDT-EDL1 was discontinued by the company in 2012 and the use of P-PDT has gradually come to an end in Japan. 

### 2.3. New Application of PDT Using Talaporfin Sodium

In 2009, PDT using a new photosensitizer, talaporfin sodium (Chemical Formula: C38H37N5Na4O9) (mono-L-aspartyl chlorin e6 [NPe6], Laserphyrin^®^; Meiji Seika Pharma Co., Ltd., Tokyo, Japan) was started for superficial carcinoma of the respiratory tract under the Japanese health insurance system [23]. Semiconductor laser light (Panasonic Healthcare Co., Ltd., Tokyo, Japan) of 664 nm was used for laser activation. PDT using talaporfin sodium (T-PDT) showed lower skin phototoxicity than P-PDT, so the period of light shielding could be shortened to within 2 weeks. Nakamura* et al.* [24] performed T-PDT and its photodynamic diagnosis for treatment in superficially infiltrated gastric cancer. T-PDT was also applied for local failure after chemoradiotherapy or radiotherapy for patients with esophageal cancer as a phase I prospective study by Yano* et al.* [25]. At present, a multi-institute phase III trial for esophageal carcinomas is underway in Japan (unpublished). Our group also started a phase I/II clinical trial of T-PDT for patients with unresectable bile duct carcinoma and resected cases with remnant cancer cells of bile duct carcinoma at the bile duct stump in 2009 [26]. At present, it is considered that T-PDT could be safely achieved, but its survival benefit remains unknown. PDT was recommended in the first edition of the Japanese guidelines of treatment for bile duct carcinoma in 2007; however, this recommendation was canceled in the second edition in 2014 [27]. 

### 2.4. Endoscopic Access of Laser Radiation for PDT

For esophageal, stomach, and intestinal carcinomas, an endoscopic approach of PDT is not difficult, but endoscopy-guided laser radiation via the bile duct is relatively difficult. The following approaches for endoscopy routes are considered: (1) the percutaneous transhepatic biliary; (2) the percutaneous transjejunal route after surgery; and (3) the endoscopic retrograde biliary route via the duodenal papilla [28]. Rumalla* et al.* [29,30] reported a route for endoscopic retrograde cholangiography using the railway method. However, the development of more precise techniques for approaching bile duct carcinoma is necessary.

## 3. Experimental Section 

### 3.1. PDT Using Talaporfin Sodium

Some investigators also showed good cancer cell toxicity in digestive tract carcinomas in an animal model [31,32]. Kasuya* et al.* [33,34] and our group also showed the increased apoptotic effects of T-PDT in a bile duct cancer cell line* in vivo* in comparison with that of P-PDT. Comparison between chemotherapy alone and PDT is expected as future work. Our basic study showed increased activity by the combination of PDT and an anti-cancer drug, such as gemcitabine or oxaliplatin [35]. 

### 3.2. PDT Using a New Photosensitizer

Tanaka* et al.* [36,37] attempted to apply PDT with the third-generation photosensitizer, glucose-conjugated chlorine (H2TFPC-SGlc), for gastric and colon carcinoma or gastrointestinal stromal tumor of the stomach in a rat model, with a specific wavelength of a semi-conductor laser. Hayashi* et al.* [38] also showed the efficacy of mannose-conjugated chlorin PDT in gastric cancer as well. Our group also examined this new agent for PDT in bile duct carcinoma* in vitro* and* in vivo* (unpublished). The development of a photosensitizer and a low-cost laser apparatus should be expected to accomplish PDT as a conventional anti-cancer therapy in digestive tract carcinoma.

## 4. Conclusions 

Although PDT is a cancer-specific and safe treatment option for digestive tract carcinomas, its clinical application has been limited worldwide, as well as in Japan. How do we move forward? Specifically, there is a need for studies regarding (1) a new photosensitizer with a more effective cytocidal effect, lower phototoxicity, and a shortened period of light shielding; (2) a portable laser apparatus with deeper penetration of tissue and lower cost; and (3) the technical development of an endoscopic approach as a future step to acquire a consensus on the conventional treatment approved by the Japanese national health insurance system.
